# Changes in Psychosocial Conditions and Eventual Mortality in Community-residing Elderly People

**DOI:** 10.2188/jea.13.72

**Published:** 2007-11-30

**Authors:** Noriyuki Nakanishi, Hideki Fukuda, Kozo Tatara

**Affiliations:** 1 Department of Social and Environmental Medicine, Course of Social Medicine, Osaka University Graduate School of Medicine.

**Keywords:** longitudinal study, mortality, elderly people, psychosocial conditions

## Abstract

We evaluated the association between changes in psychosocial conditions (assessed in 1992 and 1998) and subsequent mortality through 2001 among 741 Japanese elderly people living in a city located on Osaka in 1992. After adjustment for potential predictors of mortality, the relative risk of mortality, compared with subjects who continued to participate in social activities, was 1.44 (95% confidence interval [CI]: 0.47-4.40), 4.03 (95% CI: 2.11-7.67), and 2.31 (95% CI: 1.28-4.17) for those who started, discontinued, and did not participate at any time, respectively. The multivariate-adjusted relative risk of mortality, compared with those who did not find human relationships difficult in either survey, was 0.88 (95% CI: 0.26-3.05) for those who did not find such relationships difficult in the second survey, 1.73 (95% CI: 1.03-2.88) for those who occasionally found them difficult, and 6.62 (95% CI: 2.43-18.03) for those who continuously did so. The multivariate-adjusted relative risk of mortality, relative to those who consistently considered life worth living (*Ikigai*), was 0.72 (95% CI: 0.28-1.87), 2.22 (95% CI: 1.44-3.42), and 1.46 (95% CI: 0.65-3.31) for those who found, lost, and did not find life worth living in either survey, respectively. Deterioration in psychosocial conditions as well as continuously poor psychosocial conditions may be an important determinant of mortality risk for elderly people.

There is a growing body of knowledge to support the idea that engaging in meaningful and productive activities is a key component in promoting health and reducing the risk of mortality in later life.^1^^-^^3^ Social relationships and desirable subjective feelings are among the most studied psychosocial factors in relation to health and survival. Prospective studies have shown that older persons with a low quantity and sometimes a low quality of social relationships have an increased risk of mortality.^4^^-^^11^ From a sample of subjects living in Norway, Dalgard and Håheim^12^ determined that low social participation and, to a lesser extent, a lack of close relationships and external locus of control are associated with increased mortality after adjustment for sociodemographic and biological factors. Sugisawa et al.^13^ found in their study of elderly Japanese that social participation has a strong impact on mortality but has an indirect effect on mortality in terms of its relationship to chronic disease, functional status, and self-reported health. As for the predictive value of desirable subjective feelings, several studies in Germany, Finland, and Japan^14^^-^^17^ have found that dissatisfaction with life and lack of a sense that life is worth living (*Ikigai*) are associated with increased mortality risk and suggest that satisfaction with life and a sense that life is worth living are prognostic factors for the longevity of older people.

However, most studies^4^^-^^9^^,^^12^^-^^17^ have treated psychosocial conditions as a stable concept measured at one point in time, even though this approach gives only a snapshot of its constitution. This may be less problematic for middle-aged people, but psychosocial conditions as well as health and functional status are more dynamic among the elderly people.^18^^,^^19^ Relatively common events, such as the onset of a new illness or a sudden change in physical function, can trigger changes in psychosocial conditions for the elderly. Conversely, a change in psychosocial conditions may result in increased risk of incident disease, the precipitation of acute health events, or changes in the detection of disease. Since dynamic and reciprocal relations are likely to prevail, a cross-sectional assessment of psychosocial conditions may miss important ongoing changes in the psychosocial conditions of elderly people.

Little information is available on the association between changes in psychosocial conditions and eventual mortality,^10^^,^^11^ and whether the impact of psychosocial conditions is reflected in actual changes over time, which constitute true predictors, remains unclear. The purpose of our study was to examine the association between changes in psychosocial conditions over six years and mortality during the following three years among Japanese elderly people.

## METHODS

### Study Population

On October 1, 1992, when this study began, A city in northern Osaka Prefecture had a total population of 87,293, of whom 6,674 were aged 65 years and older. A total of 1,491 people aged 65 years and older (22.3%) were randomly selected from the computerized sex-age register, including 21 people in a nursing home. Five people in the sample were found to have died, and 13 had moved away, leaving 1,473 people to be contacted. Responses were obtained from 1,405 for a response rate of 95.4%. Reasons for nonresponse were absence (15), hospitalization (25), placement in an institution (21), and refusal to participate (7). Institutionalized people were therefore not included in this study. By the time of the follow-up 6 years later (1998), 324 (23.7%) of the 1,405 potential participants had died and 203 (14.4%) had moved away. Of the participants registered with the city hall of A in October 1998, 84.4% (741 of 878) completed a full-form, in-person interview, while the remainder either declined to be re-interviewed or underwent a proxy interview. Only the 741 participants with full-form, in-person interviews in both the baseline year and the follow-up 6 years later were included in this study. Well-trained district welfare commissioners conducted the full-form interview in the respondents’ home in both surveys. An institutional review committee approved this study. We considered the return of self-administered questionnaires signed by the subjects to imply their consent to participate in the study.

### Measurement of Psychosocial Conditions

For assessing psychosocial conditions, our questionnaire asked about elderly people’s involvement in social networks and participation in activities considered to be particularly meaningful. Details of the questionnaire have been described elsewhere.^17^^,^^20^ The questions in the questionnaire were worded as follows: ‘Are you participating in social activities?’, ‘Do you find relationships with people outside the family or with members of the family difficult?’ and ‘Do you have *Ikigai*?’ which could be translated directly as ‘Do you have anything to live for?’ *Ikigai* refers to particular aspects of one’s life which make it meaningful or worth living.^21^ The replies obtained in both the baseline year (1992) and in the 1998 follow-up survey were in response to questions using identical wording. Each of the psychosocial conditions in 1992 was cross-tabulated with that in 1998, and four stability/change-variables were constructed as the independent variables for the analyses.

### Covariates

We considered several demographic, health and functional status measures as covariates in the multivariate modeling process. The demographic characteristics covered sex and age. We inquired into day-to-day preventive health practices related to diet and exercise and into current medical treatment. For assessing functional status, we adopted the concepts and methods developed by the Social Survey Division of the Office of Population Censuses^22^ designed to measure ten main areas or types of ability or disability: locomotion, reaching and stretching, dexterity, seeing, hearing, personal care, continence, communication, behavior, and intellectual functioning. Each area is scored on a scale from 0, which represents no disability, up to the maximum, which differs for each item, with the highest number corresponding to the most severe grade of disability. Because multiple disabilities are frequent and different disabilities may vary in severity, the Office of Population Censuses survey developed an overall individual severity score based on a formula using the scores for the three worst disability scores: worst + 0.4 (second worst) + 0.3 (third worst). In our study, we also adopted this method to arrive at the Overall Disability Score, and disability was defined as an overall severity score of 5 or more. Presence of disability (none vs any) was assessed both at the baseline (1992) and at the 1998 follow-up.

### Cohort Follow-up and Statistical Analyses

The survey population’s status as of the end of October 2001 was determined from their resident registration cards and death certificates to verify their eligibility and outcomes. Of the 741 subjects enrolled in this study, the follow-up could be completed for only 704 (112 deceased and 592 alive) because 37 had moved away by the time of the follow-up. For each participant, person-years of follow-up were calculated by using the date of the 1998 interview and the date of death or the date of the follow-up (end of October 2001), or the date of the last registration. Those who had moved away during the follow-up period were assigned censored survival times as were those members of the cohort who were still residing in A city at the end of the follow-up. The follow-up rate was 98.4% of the total potential follow-up person-years. Cox’s proportional hazards model^23^ was used to evaluate the association between changes in psychosocial conditions (assessed in 1992 and 1998) and subsequent mortality through 2001. Data were adjusted for age, sex, changes in disability, daily preventive health services, medical treatment, and changes in all other psychosocial conditions.

Data were analyzed with the SPSS/PC^®^ statistical package (SPSS Inc., Chicago, Illinois). All reported p values are two-tailed and those less than 0.05 were considered statistically significant.

## RESULTS

Table 1 shows characteristics of the study population according to age. As for changes in participation in social activities from 1992 to 1998, 31.3% of the subjects continued to participate in social activities, 9.0% started, 14.4% discontinued, and 45.2% did not participate at the time of either survey. The percentage of those who continued to participate in social activities decreased with age, and the proportion of those who did not participate at the time of either survey increased. As for changes in difficulties with human relationships, 86.1% of the subjects did not find such relationships difficult at the time of either survey, 3.1% did not find relationships with people difficult at the second survey, 9.6% found them difficult in 1992, and 1.2% reported difficulties at the time of both surveys. The proportion of those who did not have difficulties with human relationships at the time of either survey decreased with age, and 20.6% of those aged 77 to 92 years found them difficult in 1998. As for changes in the concept of a life worth living (*Ikigai*), 50.9% of the subjects found life worth living in both surveys, 7.4% found life worth living in 1998 only, 34.7% lost their sense that life was worth living, and 7.0% did not have any such concept at the time of either survey. Changes in the concept of a life worth living did not differ significantly among the three age groups. As for changes in disability, 60.3% of the subjects did not have any disability at the time of either survey, 2.3% did not have any disability at the second survey, 25.8% had some disability in 1998, and 11.6% at the time of both surveys. The percentage of those who did not have any disability in either survey deceased with age, and the percentage of those who had some disability at the second survey increased. Two-thirds of the subjects were female, and the percentage of women was the highest among those aged 71 to 76 years. The percentage of those who were engaged in daily health promotional practices in 1998 was 77.3%, and decreased with age. The proportion of those who were receiving medical treatment in 1998 was 85.3%, and there were no significant differences in medical treatment among the three age groups.

**Table 1.** Characteristics of the study population by age group.

Variable	Age (1992)								p value

	65-70 years		71-76 years		77-92 years		Total		
	(n=326)		(n=260)		(n=155)		(n=741)		
	no. of the elderly (% of group)								
Participation in social activities, 1992 and 1998									
Yes, yes	123	(37.7)	84	(32.3)	25	(16.1)	232	(31.3)	< 0.001
No, yes	39	(12.0)	18	(6.9)	10	(6.5)	67	(9.0)	
Yes, no	37	(11.3)	46	(17.7)	24	(15.5)	107	(14.4)	
No, no	127	(39.0)	112	(43.1)	96	(61.9)	335	(45.2)	
Finding relationships with people difficult, 1992 and 1998									
No, no	297	(91.1)	224	(86.2)	117	(75.5)	638	(86.1)	< 0.001
Yes, no	6	(1.8)	15	(5.8)	2	(1.3)	23	(3.1)	
No, yes	23	(7.1)	16	(6.2)	32	(20.6)	71	(9.6)	
Yes, yes	0	(0.0)	5	(1.9)	4	(2.6)	9	(1.2)	
Life worth living (*Ikigai*), 1992 and 1998									
Yes, yes	170	(52.1)	128	(49.2)	79	(51.0)	377	(50.9)	0.382
No, yes	27	(8.3)	16	(6.2)	12	(7.7)	55	(7.4)	
Yes, no	111	(34.0)	90	(34.6)	56	(36.1)	257	(34.7)	
No, no	18	(5.5)	26	(10.0)	8	(5.2)	52	(7.0)	
Sex									
Female	197	(60.4)	193	(74.2)	105	(67.7)	495	(66.8)	0.002
Male	129	(39.6)	67	(25.8)	50	(32.3)	246	(33.2)	
Disability, 1992 and 1998									
None, none	253	(77.6)	149	(57.3)	45	(29.0)	447	(60.3)	< 0.001
Some, none	0	(0.0)	10	(3.8)	7	(4.5)	17	(2.3)	
None, some	50	(15.3)	81	(31.2)	60	(38.7)	191	(25.8)	
Some, some	23	(7.1)	20	(7.7)	43	(27.7)	86	(11.6)	
Daily health promotional practices, 1998									
Yes	278	(85.3)	205	(78.8)	90	(58.1)	573	(77.3)	< 0.001
No	48	(14.7)	55	(21.2)	65	(41.9)	168	(22.7)	
Receiving medical treatment, 1998									
No	57	(17.5)	34	(13.1)	18	(11.6)	109	(14.7)	0.154
Yes	269	(82.5)	226	(86.9)	137	(88.4)	632	(85.3)	

Table 2 shows univariate survival results for psychosocial conditions and other covariates. As for change in participation in social activities from 1992 to 1998, the relative risk of mortality, compared with the risk for subjects who continued to participate in social activities from 1992 until 1998, was 0.85 (95% confidence interval [CI]: 0.29-2.55) for those who started participating in such activities during the survey period, 4.62 (95% CI: 2.50-8.54) for those who discontinued, and 2.91 (95% CI: 1.68-5.03) for those who did not participate in social activities at any time. As for changes in difficulties with human relationships, the relative risk of mortality, compared with the risk for those who did not find relationships with people difficult at the time of either survey, was 1.10 (95% CI: 0.35-3.49) for those who did not find such relationships difficult in 1998, 3.57 (95% CI: 2.29-5.57) for those who found them difficult in 1998, and 9.15 (95% CI: 3.97-21.07) for those who continued to find them difficult throughout the 6-year period. As for changes in the concept of a life worth living (*Ikigai*), the relative risk of mortality, relative to the risk for those who continuously found life worth living, was 0.97 (95% CI: 0.38-2.47) for those who had started to find life worth living at the second survey, 2.55 (95% CI: 1.70-3.83) for those who lost their sense of a life worth living, and 1.78 (95% CI: 0.86-3.69) for those who did not have any such concept at the time of either survey. Sex, age, changes in disability, and daily health promotional practices were significantly associated with the risk for mortality, whereas medical treatment showed only a moderate association. We considered these variables to be potentially confounding variables for determining the association of psychosocial conditions with mortality.

**Table 2.** Univariate survival results for psychosocial conditions and other covariates.

Variable	Number that died	Total person-years	Rate per 1000 person-years	Relative risk (95% CI)
Participation in social activities, 1992 and 1998				
Yes, yes	16	685	23.4	1.00
No, yes	4	201	19.9	0.85 (0.29-2.55)
Yes, no	28	267	105.0	4.62 (2.50-8.54)
No, no	64	955	67.0	2.91 (1.68-5.03)
Finding relationships with people difficult, 1992 and 1998				
No, no	77	1849	41.6	1.00
Yes, no	3	65	45.9	1.10 (0.35-3.49)
No, yes	26	176	147.3	3.57 (2.29-5.57)
Yes, yes	6	17	352.1	9.15 (3.97-21.07)
Life worth living (*Ikigai*), 1992 and 1998				
Yes, yes	38	1111	34.2	1.00
No, yes	5	150	33.3	0.97 (0.38-2.47)
Yes, no	60	698	85.9	2.55 (1.70-3.83)
No, no	9	148	60.9	1.78 (0.86-3.69)
Sex				
Female	57	1431	39.8	1.00
Male	55	677	81.3	2.07 (1.43-2.99)
Age, 1992				
65-70 years	21	962	21.8	1.00
71-76 years	38	733	51.8	2.39 (1.40-4.07)
77-92 years	53	413	128.4	6.01 (3.62-9.96)
Disability, 1992 and 1998				
None, none	37	1295	28.6	1.00
Some, none	2	46	44.0	1.55 (0.37-6.44)
None, some	46	533	86.4	3.04 (1.98-4.69)
Some, some	27	235	115.1	4.12 (2.51-6.76)
Daily health promotional practices, 1998				
Yes	75	1649	45.5	1.00
No	37	458	80.7	1.79 (1.21-2.65)
Receiving medical treatment, 1998				
No	12	314	38.2	1.00
Yes	100	1794	55.7	1.46 (0.80-2.65)

CI: confidence interval

Table 3 shows the age-adjusted and multivariate-adjusted mortality risks according to changes in psychosocial conditions. Relative risks of mortality were reduced for each psychosocial condition after adjustment for age and with further adjustment for potentially confounding factors in the full model. When we evaluated the associative changes in psychosocial conditions and the risk of mortality in the fully adjusted model, the mortality risk, compared with the risk for subjects who continued to participate in social activities, was 1.44 (95% CI: 0.47-4.40) for those who began participating during the survey period, 4.03 (95% CI: 2.11-7.67) for those who discontinued participation, and 2.31 (95% CI: 1.28-4.17) for those who did not participate in social activities at the time of either survey. The multivariate-adjusted relative risk of mortality, compared with the risk for those who did not find relationships with people difficult at the time of either survey, was 0.88 (95% CI: 0.26-3.05) for those who did not find such relationships difficult in 1998, 1.73 (95% CI: 1.03-2.88) for those who found them difficult in 1998, and 6.62 (95% CI: 2.43-18.03) for those who continued to find them difficult throughout the survey period. As for changes in the concept of a life worth living (Ikigai), the multivariate-adjusted relative risk of mortality, relative to the risk for those who continued to find life worth living, was 0.72 (95% CI: 0.28-1.87) for those who found life worth living in 1998, 2.22 (95% CI: 1.44-3.42) for those who lost their sense of a life worth living, and 1.46 (95% CI: 0.65-3.31) for those who did not find life worth living at the time of either survey.To assess the effect of changes in disability on the association between changes in psychosocial conditions and the risk of mortality, we studied the relation between changes in psychosocial conditions and the risk of mortality according to changes in disability (Table 4). For this analysis, the subjects were divided into two groups based on the presence of disability at the second survey. We also combined the subjects who started to participate in social activities with those who continued to participate in social activities, those did not find relationships with people difficult at the second survey with those did not find such relationships difficult at the time of either survey, and those who found life worth living in 1998 only with those who found life worth living throughout the survey period. Among the subjects who did not have any disability at the time of the second survey, the multivariate-adjusted mortality risk, compared with the risk for those who continued or started to participate in social activities, was 1.71 (95% CI: 0.60-4.87) for those who discontinued participation in social activities and 2.61 (95% CI: 1.20-5.70) for those who did not participate at the time of either survey. The multivariate-adjusted relative risk of mortality, compared with the risk for those who did not find relationships with people difficult at either survey or at the second survey, was 5.83 (95% CI: 1.23-27.60) for those who found them difficult in 1998. As for changes in the concept of a life worth living (*Ikigai*), the multivariate-adjusted relative risk of mortality, relative to the risk for those who continued to find life worth living or found life worth living, was 5.61 (95% CI: 2.70-11.65) for those who lost their sense that life was worth living and 0.65 (95% CI: 0.08-5.40) for those who did not find life worth living at the time of either survey. Among the subjects who had some disability at the second survey, the multivariate-adjusted mortality risk, compared with the risk for those who continued or started to participate in social activities, was 8.13 (95% CI:3.50-18.89) for those who discontinued participation and 2.34 (95% CI: 1.10-4.50) for those who did not participate in social activities at the time of either survey. The multivariate-adjusted relative risk of mortality, compared with the risk for those who did not find relationships with people difficult at either survey or at the second survey, was 1.84 (95% CI: 11.05-3.23) for those who found them difficult in 1998 and 8.72 (95% CI: 3.02-25.18) for those who continued to find relationships with people difficult. The multivariate-adjusted relative risk of mortality, relative to the risk for those who continued to find life worth living or found life worth living in 1998, was 1.82 (95% CI: 1.05-3.13) for those who lost their sense that life was worth living and 3.77 (95% CI: 1.45-9.78) for those who did not find life worth living at the time of either survey.

**Table 3.** Age-adjusted and multivariate-adjusted mortality risk according to changes in psychosocial conditions.

Variable	Age-adjusted relative risk (95% CI)	Multivariate-adjusted relative risk (95% CI)*
Participation in social activities, 1992 and 1998		
Yes, yes	1.00	1.00
No, yes	0.81 (0.27-2.42)	1.44 (0.47-4.40)
Yes, no	3.68 (1.98-6.84)	4.03 (2.11-7.67)
No, no	2.14 (1.23-3.74)	2.31 (1.28-4.17)
Finding relationships with people difficult, 1992 and 1998		
No, no	1.00	1.00
Yes, no	1.04 (0.33-3.31)	0.88 (0.26-3.05)
No, yes	2.67 (1.69-4.23)	1.73 (1.03-2.88)
Yes, yes	6.23 (2.68-14.49)	6.62 (2.43-18.03)
Life worth living (*Ikigai*), 1992 and 1998		
Yes, yes	1.00	1.00
No, yes	1.00 (0.39-2.54)	0.72 (0.28-1.87)
Yes, no	2.55 (1.70-3.83)	2.22 (1.44-3.42)
No, no	1.85 (0.89-3.83)	1.46 (0.65-3.31)

* Adjusted for sex, age, change in disability, daily health promotional practices, medical treatment, and changes in all other psychosocial conditions.

CI: confidence interval

**Table 4.** Age-adjusted and multivariate-adjusted mortality risk according to changes in psychosocial conditions and disability.

Disability, 1992 and 1998 Variable	Age-adjusted relative risk (95% CI)	Multivariate-adjusted relative risk (95% CI)*
None or some, none		
Participation in social activities, 1992 and 1998		
Yes or no, yes	1.00	1.00
Yes, no	2.72 (1.00-7.39)	1.71 (0.60-4.87)
No, no	2.53 (1.22-5.26)	2.61 (1.20-5.70)
Finding relationships with people difficult, 1992 and 1998		
No or yes, no	1.00	1.00
No, yes	3.09 (0.74-12.96)	5.83 (1.23-27.60)
Life worth living (*Ikigai*), 1992 and 1998		
Yes or no, yes	1.00	1.00
Yes, no	5.65 (2.80-11.41)	5.61 (2.70-11.65)
No, no	1.00 (0.13-7.80)	0.65 (0.08-5.40)
None or some, some		
Participation in social activities, 1992 and 1998		
Yes or no, yes	1.00	1.00
Yes, no	3.40 (1.57-7.39)	8.13 (3.50-18.89)
No, no	1.73 (0.84-3.59)	2.34 (1.10-4.50)
Finding relationships with people difficult, 1992 and 1998		
No or yes, no	1.00	1.00
No, yes	2.06 (1.24-3.43)	1.84 (1.05-3.23)
Yes, yes	7.09 (2.95-17.03)	8.72 (3.02-25.18)
Life worth living (*Ikigai*), 1992 and 1998		
Yes or no, yes	1.00	1.00
Yes, no	1.62 (0.99-2.65)	1.82 (1.05-3.13)
No, no	1.92 (0.87-4.22)	3.77 (1.45-9.78)

* Adjusted for sex, age, change in disability, daily health promotional practices, medical treatment, and changes in all other psychosocial conditions.

CI: confidence interval

## DISCUSSION

In this study, both sustained poor social relations and a decline in the level of social relations (assessed by participation in social activities and finding relationships with people difficult) over a 6-year period, compared with sustained high levels, remained statistically significant factors associated with mortality when adjusted for potential predictors of mortality. Lack of a sense that life was worth living during the follow-up period, relative to a sustained *Ikigai*, was also independently associated with an increased mortality risk. In addition, an improvement in psychosocial conditions was associated with a mortality risk similar to that for persons with sustained high levels of psychosocial conditions. Analysis of mortality risk according to the presence of disability at the second survey revealed that discontinuation of social participation had a higher association with the subsequent mortality risk for those who had some disability than for those who did not. On the other hand, finding relationships with people difficult and lack of a sense that life was worth living showed a stronger correlation with the subsequent mortality risk for those who did not have any disability than for those who did. Although a decline in functional status may have caused discontinuation of social participation and thus could have increased the risk of mortality, our results indicate that deterioration in psychosocial conditions as well as sustained poor psychosocial conditions may be an independent determinant of mortality risk for elderly people. Our results also imply that assessment of changes in psychosocial conditions may be another important predictive factor for the mortality risk for elderly people.

The mechanism of how psychosocial conditions influence or affect health outcomes remains unclear. Silverstein and Bengtson^24^ have suggested psychobiological and social behavioral factors to explain the link between social relationships and health. Some investigators1,^24^^-^^27^ have found that a supportive social environment increases the ability to resist disease and other environmental insults via its effects on physiological processes such as the immune and neuroendocrine functions. Nakanishi et al.^7^ have demonstrated close relationships between participation in social activities and the use of health checks and daily health promotional practices. Not only many social relationships affect health because they are or are not supportive, they may also regulate or control human thought, feeling and behavior in ways that promote health.^28^^,^^29^ Furthermore, Nakanishi et al.^7^^,^^21^ have indicated that identification of particular aspects of life (*Ikigai*) may reflect an active physiological and psychological profile and encourage participation in social activities. Sustaining and developing high levels of social relations and finding life worth living appear to have generally beneficial effects on health, not solely or even primarily attributable to their buffering effects, and there may be aspects of social relationships other than their supportive quality that account for these effects. The finding that persons whose social relationships improved had mortality risks similar to those with sustained high levels of social relationships also raises the possibility of a buffering effect of an increase in psychosocial conditions that may be tapped in times of rapid changes in health status. We clearly need a better understanding of the biological, social, and psychological processes that link the existence, quantity, structure, or content of psychosocial conditions to health.

There are several limitations to our study. First, we studied the relation between changes in psychosocial conditions and the risk of mortality according to changes in disability. Our study design, however, could not determine accurately whether changes in psychosocial conditions preceded, occurred simultaneously with, or followed changes in health and functional status. It therefore remains to be determined whether deterioration in psychosocial conditions precipitates changes in health status (and therefore greater mortality risk) or vice versa, which would make psychosocial conditions merely a proxy for failing health. Second, the cohort of this study was selected from among a vigorous older group showing a ‘healthy older status’. Since the majority of the elderly people susceptible to poor psychosocial conditions may have died prior to the age of the cohort, this would leave only people who are relatively immune to changes in psychosocial conditions. Third, the studies that have considered the sex and/or age groups separately have shown some inconsistent results for gender and age differences in the strength and direction of the relationship between social relations and mortality risk.^5^^,^^11^ Such findings call into question the desirability of treating sex and age merely as control variables in research on this subject. In fact, they seem to indicate the need to construct different models for this relationship for men and women, as well as for older and younger individuals. Further more detailed investigations are thus needed to clarify the theoretical causal mechanisms of specific associations of changes in psychosocial conditions with mortality. Our findings, based on a community-residing elderly population, suggest that the deterioration in psychosocial conditions as well as sustained poor psychosocial condition may be independently associated with mortality. One of the key conceptual issues which the association of psychosocial conditions and mortality raises is whether psychosocial conditions are merely a marker for general decline or play a causative role. If there is a causal association, it may be possible to anticipate and compensate for those situations in which deteriorating psychosocial conditions result in untoward consequences.

## References

[1] Cassel J. Psychosocial processes and “stress”: theoretical formulation. Int J Health Serv 1974;4:471-82. 4475665 10.2190/WF7X-Y1L0-BFKH-9QU2

[2] House JS, Landis KR, Umberson D. Social relationships and health. Science 1988;241:540-5. 3399889 10.1126/science.3399889

[3] Rowe JR, Kahn RL. Successful aging. New York: Pantheon Books, 1998.

[4] Welin L, Tibblin G, Svärdsudd K, Tibblin B, Ander-Peciva S, Larsson B, . Prospective study of social influences on mortality. The study of men bom in 1913 and 1923. Lancet 1985;1:915-8. 2858755 10.1016/s0140-6736(85)91684-8

[5] Schoenbach VJ, Kaplan BH, Fredman L, Kleinbaum DG. Social ties and mortality in Evans County, Georgia. Am J Epidemiol 1986;123:577-91. 3953538 10.1093/oxfordjournals.aje.a114278

[6] Avlund K, Damsgaard MT, Holstein BE. Social relations and mortality. An eleven year follow-up study of 70-year-old men and women in Denmark. Soc Sci Med 1998;47:635-43. 9690846 10.1016/s0277-9536(98)00122-1

[7] Nakanishi N, Tatara K. Correlates and prognosis in relation to participation in social activities among older people living in a community in Osaka, Japan. J Clin Geropsychol, 2000;6:299-307.

[8] Lennartsson C, Silverstein M. Does engagement with life enhance survival of elderly people in Sweden? The role of social and leisure activities. J Gerontol B Psychol Sci Soc Sci 2001;56:S335-42. 11682594 10.1093/geronb/56.6.s335

[9] Ceria CD, Masaki KH, Rodriguez BL, Chen R, Yano K, Curb JD. The relationship of psychosocial factors to total mortality among older Japanese-American men: the Honolulu Heart Program. J Am Geriatr Soc 2001;49:725-31. 11454110 10.1046/j.1532-5415.2001.49148.x

[10] Cerhan JR, Wallace RB. Change in social ties and subsequent mortality in rural elders. Epidemiology 1997;8:475-81. 9270946 10.1097/00001648-199709000-00001

[11] Lund R, Modvig J, Due P, Holstein BE. Stability and change in structural social relations as predictor of mortality among elderly women and men. Eur J Epidemiol 2000; 16:1087-97. 11484796 10.1023/a:1010897810514

[12] Dalgard OS, Håheim LL. Psychosocial risk factors and mortality: a prospective study with special focus on social support, social participation, and locus of control in Norway. J Epidemiol Community Health 1998;52:476-81. 9876357 10.1136/jech.52.8.476 PMC1756754

[13] Sugisawa H, Liang J, Liu X. Social networks, social support, and mortality among older people in Japan. J Gerontol 1994;49:S3-13. 8282987 10.1093/geronj/49.1.s3

[14] Maier H, Smith J. Psychological predictors of mortality in old age. J Gerontol B Psychol Sci Soc Sci 1999;54:P44-54. 9934395 10.1093/geronb/54b.1.p44

[15] Koivumaa-Honkanen H, Honkanen R, Viinamäki H, Heikkilä K, Kaprio J, Koskenvuo M. Self-reported life satisfaction and 20-year mortality in healthy Finnish adults. Am J Epidemiol 2000;152:983-91. 11092440 10.1093/aje/152.10.983

[16] Nakanishi N, Tatara K, Tatatorige T, Murakami S, Shinsho F. Effects of preventive health services on survival of the elderly living in a community in Osaka, Japan. J Epidemiol Community Health 1997;51:199-204. 9196652 10.1136/jech.51.2.199 PMC1060445

[17] Nakanishi N, Tatara K, Shinsho F, Murakami S, Takatorige T, Fukuda H, . Mortality in relation to urinary and faecal incontinence in elderly people living at home. Age Ageing 1999;28:301-6. 10475868 10.1093/ageing/28.3.301

[18] Berkman LF. The assessment of social networks and social support in the elderly. J Am Geriatr Soc 1983;31:743-9. 6655175 10.1111/j.1532-5415.1983.tb03393.x

[19] Minkler M. Social support and health of the elderly. In: Cohen S, Syme SL (eds.), Social Support and Health. New York: Academic Press, 1985:199-216.

[20] Nakanishi N, Tatara K, Naramura H, Fujiwara H, Takashima Y, Fukuda H. Urinary and fecal incontinence in a community-residing older population in Japan. J Am Geriatr Soc 1997;45:215-9. 9033523 10.1111/j.1532-5415.1997.tb04511.x

[21] Nakanishi N. ‘Ikigai’ in older Japanese people. Age Ageing 1999;28:323-4. 10475874 10.1093/ageing/28.3.323

[22] Martin J, Meltzer H, Elliot D. The prevalence of disability among adults. OPCS Surveys of Disability in Great Britain, Report 1. London: Her Majestry’s Stationary Office, 1988.

[23] Cox DR. Regression models and life-tables (with discussion). J Royal Stat Soc, Series B, 1972;34:187-220.

[24] Silverstein M, Bengtson VL. Do close parent-child relations reduce the mortality risk of older parents? J Health Soc Behav 1991;32:382-95. 1765628

[25] Jemmott JB 3rd, Locke SE. Psychosocial factors, immunologic mediation, and human susceptibility to infectious diseases: how much do we know? Psychol Bull 1984;95:78-108. 6544433

[26] Kaplan GA, Roberts RE, Camacho TC, Coyne JC. Psychosocial predictors of depression. Prospective evidence from the human population laboratory studies. Am J Epidemiol 1987;125:206-20. 3812429 10.1093/oxfordjournals.aje.a114521

[27] Kiecolt-Glaser JK, Glaser R, Williger D, Stout J, Messick G, Sheppard S, . Psychosocial enhancement of immunocompetence in a geriatric population. Health Psychol 1985;4:25-41. 2990890 10.1037//0278-6133.4.1.25

[28] Antonovsky A. Health, Stress and Coping. San Francisco: Jossey-Bass, 1979.

[29] Umberson D. Family status and health behaviors: social control as a dimension of social integration. J Health Soc Behav 1987;28:306-19. 3680922

